# Präklinische perimortale Crash-Sectio im Rahmen einer Traumareanimation: Fallbericht mit Darstellung gewonnener Erkenntnisse

**DOI:** 10.1007/s00113-022-01220-w

**Published:** 2022-08-10

**Authors:** Justus Wolff, Florian Breuer, Konrad von Kottwitz, Stefan Poloczek, Tom Röschel, Janosch Dahmen

**Affiliations:** 1 Charité Universitätsmedizin Berlin, Berlin, Deutschland; 2 Ärztliche Leitung Rettungsdienst Rheinisch-Bergischer Kreis, Amt für Feuerschutz und Rettungswesen, Bergisch Gladbach, Deutschland; 3 Notfallzentrum, Unfallkrankenhaus Berlin, Berlin, Deutschland; 4 Ärztliche Leitung Rettungsdienst Berlin, Berliner Feuerwehr, Berlin, Deutschland; 5 Klinik für Anästhesiologie, Intensiv- und Schmerzmedizin, Unfallkrankenhaus Berlin, Berlin, Deutschland; 6 Fakultät für Gesundheit, Department Humanmedizin, Universität Witten/Herdecke, Witten/Herdecke, Deutschland

**Keywords:** Reanimation, Schwangerschaft, Perimortaler Kaiserschnitt, Traumatisch bedingter Kreislaufstillstand, Rettungsdienst, Cardiopulmonary resuscitation, Pregnancy, Perimortem cesarean section, Traumatic cardiac arrest, Emergency medical services

## Abstract

Es wird über eine präklinische Crash-Sectio bei einer schwangeren Patientin im traumatischen Herz-Kreislauf-Stillstand nach Fenstersturz berichtet. Die für die Präklinik gewonnenen Erkenntnisse der Einsatznachbereitung zu diesem Fall sollen vor dem Hintergrund der aktuellen ERC-Guidelines dargelegt und ein Literaturüberblick gegeben werden. Trotz prolongierter und umfassender Maßnahmen entlang aktueller Leitlinienempfehlungen verstarben Mutter und Kind noch am Einsatzort. Die Traumareanimation einer schwangeren Patientin, einschließlich Durchführung einer präklinischen perimortalen Crash-Sectio, stellt ein sehr seltenes und anspruchsvolles wie gleichermaßen emotional belastendes Einsatzszenario für alle Beteiligten dar.

## Abstract (en)

The following case report discusses the resuscitation of a pregnant woman in traumatic cardiac arrest after a fall from a height with consecutive resuscitative hysterotomy for maternal and fetal salvage. The report illustrates all lessons learned from critical appraisal amid new guideline recommendations and gives an overview of the published literature on the matter. Despite extensive resuscitation efforts, ultimately both the mother and the newborn were pronounced life extinct at the scene. Prehospital treatment of (traumatic) cardiac arrest in a pregnant patient as well as performing a perimortem cesarean section remain infrequent but challenging scenarios.

## Einleitung

Das Vorgehen bei der präklinischen Reanimation von schwangeren Patientinnen wie auch die Anwendung maximalinvasiver Maßnahmen unter (präklinischer) Reanimation schwer verletzter Patienten werden kontrovers diskutiert. In den aktuellen Reanimationsleitlinien des ERC wird im Falle eines Kreislaufstillstandes in der Schwangerschaft ab der 20. Schwangerschaftswoche (SSW) und 4 min Reanimationsdauer eine unverzügliche perimortale Crash-Sectio zur Entbindung des Neugeborenen empfohlen [11]. Als präklinische perimortale Crash-Sectio (PPCS) wird der perimortale Kaiserschnitt („perimortal cesarean section“ [PMCS]) im prähospitalen Setting bezeichnet. Das Überleben von Mutter und Kind nach präklinisch durchgeführter perimortaler Crash-Sectio ist auch nach prolongierter Versorgungszeit in der Literatur beschrieben [17]. Das Überleben nach präklinisch durchgeführter Traumareanimation, einschließlich Anwendung maximalinvasiver Interventionen, wird mit bis zu 12 % angegeben [16].

**Figure 1:**
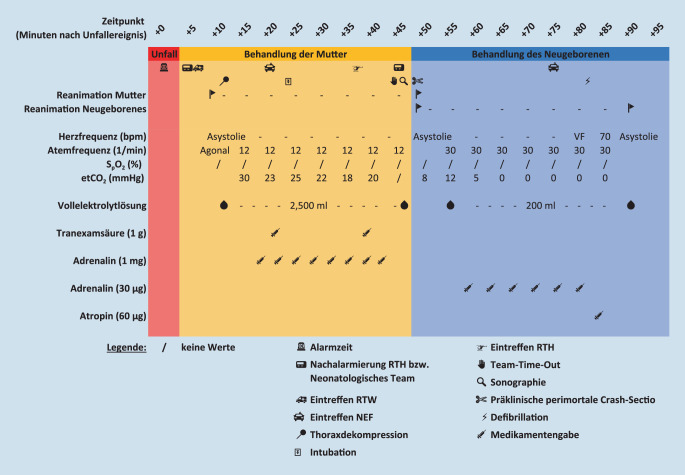

Der vorliegende Fallbericht und Literaturüberblick sowie die im Anschluss an den Einsatz erarbeitete und hier vorgestellte medizinische Handlungsanweisung (SOP) fokussieren sich auf die prähospitale Versorgung schwangerer Patientinnen im Kreislaufstillstand und die Möglichkeiten und Grenzen der PPCS. Das Manuskript orientiert sich hierbei an den Empfehlungen der „case report guidelines“ [8] und den Empfehlungen zur Erstellung systematischer Literaturreviews [12]. Alle Zeitangaben/Zeitpunkte in Klammern beziehen sich auf den mutmaßlichen Unfallzeitpunkt. Um die entsprechenden Zeitangaben möglichst exakt zu rekonstruieren, wurden mehrere Quellen genutzt (u. a. Rettungsdienstprotokolle, Leitstellendokumentation, persönliche Aufzeichnungen). Daher sind die Angaben teils mit Unschärfe assoziiert. Soweit nicht anders bezeichnet, sind sämtliche Zeitangaben in Minuten nach Unfallzeitpunkt angegeben. Der zeitliche Verlauf des Einsatzes wurde darüber hinaus tabellarisch aufgearbeitet und grafisch dargestellt (Abb. 1).

## Anamnese

An einem Spätsommernachmittag stürzte eine im 8. Monat schwangere junge Frau aus einem Fenster im 4. Stock (ca. 12 m) einer Gemeinschaftsunterkunft (+0 min). Unmittelbar nach dem beobachteten Sturz erfolgten mehrere Notrufe, aus denen sich im Ergebnis der standardisierten Notrufabfrage (SNAP) der Feuerwehrleitstelle, bedingt durch Sprachbarrieren und Mehrfachanrufe, ein uneinheitliches Lagebild ergab. Die Erstalarmierung zur Einsatzstelle in einen Außenbezirk Berlins erfolgte zum SNAP-Code „17D01P – Sturz aus extremer Höhe (mehr als 10 m) im öffentlichen Raum“ zum Einsatzstichwort „TH 1 + NOTF 2 [NA]“ [13]. Aufgrund dieses Meldebildes wurden durch die Leitstelle initial 2 Lösch- und Hilfeleistungsfahrzeuge (LHF), 2 Rettungswagen (RTW), ein Notarzteinsatzfahrzeug (NEF) sowie ein organisatorischer Leiter Rettungsdienst (OrgL RD) disponiert (+2 min). Aufgrund der Einsatzbeschreibung wurde zusätzlich der diensthabende Oberarzt vom Dienst (OAvD) der Berliner Notfallrettung, der zugleich auch die Funktion des leitenden Notarztes (LNA) wahrnimmt, zur Unterstützung alarmiert (+5 min).

Noch auf der Anfahrt erfolgte durch einen der alarmierten RTW aufgrund der abgelegenen Lage der Einsatzstelle die Nachforderung eines Rettungshubschraubers (RTH/ITH) (+7 min). Bei Eintreffen der ersten Rettungskräfte am Einsatzort (+8 min) wurde die Patientin im Hinterhof auf dem Boden liegend bewusstlos (GCS 3) mit agonaler Atmung vorgefunden. Fremdanamnestisch berichteten Zeugen über eine bis zum Unfallereignis gesunde und in der 32. SSW schwangere Patientin, bei der nach dem beobachteten Sturz bis kurz vor Eintreffen der ersten Einsatzkräfte noch Lebenszeichen (Extremitätenbewegungen und verbale Reaktionen) zu beobachten gewesen sein sollen.

## Befund

Nach Eintreffen der Einsatzkräfte des ersteintreffenden RTW bei der Patientin wurde die sofortige kardiopulmonale Reanimation entsprechend den Standard Operating Procedures der Berliner Notfallrettung, angelehnt an den ERC-Algorithmus, eingeleitet. Mit Eintreffen des ersten NEF an der Einsatzstelle (+23 min) erfolgte die erweiterte Versorgung mit dem Ziel der Beseitigung reversibler Ursachen:

- A: orotracheale First-Pass-Intubation (7,5 mm ID) nach initialer Beatmung über supraglottischen Atemweg, HWS-Immobilisierung,
- B: Nach bereits erfolgten beidseitigen Entlastungspunktionen nach Monaldi, anschließende Thoraxdrainagenanlage beidseits in Bülau-Position. Linksseitig Entleerung von Blut, rechtsseitig Entlastung eines Spannungspneumothorax. Nach Entlastung vesikuläres Atemgeräusch beidseits, etCO_2_ unter laufender Reanimation zunächst bei 30 mm Hg, im Verlauf abfallend, S_p_O_2_ zu keinem Zeitpunkt messbar.
- C: keine relevanten externen Blutungen, deutliche Instabilität des Beckens, hier sofortige Anlage einer Beckenschlinge. Seit der ersten Rhythmusanalyse anhaltend Asystolie. Analog ERC-ALS-Algorithmus repetitive Gabe von Adrenalin via i.o.-Zugang an der proximalen Tibia linksseitig. Infusion von vorgewärmter Vollelektrolytlösung mit hoher Laufrate und 1 g Tranexamsäure.

## Verlauf

Nach Eintreffen eines RTH (im Land Berlin ein Rettungsmittel mit zusätzlicher Expertise u. a. in maximalinvasiven Maßnahmen) an der Einsatzstelle (+38 min) unterstützte dieses Team die laufenden Reanimationsmaßnahmen [3]. Zur weiteren Volumentherapie wurden zwei großlumige periphervenöse Zugänge etabliert und die Gabe von 1 g Tranexamsäure wiederholt. Bei anhaltender Reanimationspflichtigkeit erfolgte nun ein Team-Time-Out („10 for 10“) unter Einbeziehung aller Einsatzkräfte (+45 min). Als reversible Ursachen des traumatischen Kreislaufstillstandes wurden bis zu diesem Zeitpunkt die Hypoxie, der Spannungspneumothorax und, soweit beim stumpfen Trauma prähospital möglich, die Hypovolämie durch Gabe von Infusionslösung und Tranexamsäure sowie Anlage einer Beckenschlinge behandelt. Als mögliche weitere Maßnahmen zur Adressierung reversibler Ursachen nach traumatischem Kreislaufstillstand verblieben die Entlastung einer potenziellen Perikardtamponade und die Reduktion der aortokavalen Kompression durch den Fetus. Eine sodann durchgeführte fokussierte Sonographie (+47 min) zum Ausschluss einer Perikardtamponade brachte aufgrund eines massiven Pneumoperitoneums und subkutanen Emphysems keine verwertbaren Befunde. Eine eindeutige Indikation für eine Clamshell-Thorakotomie bestand aufgrund der verstrichenen Zeit („elapsed time“) von mehr als 15 min bei stumpfem Traumamechanismus nicht mehr. Wegen des zu diesem Zeitpunkt bereits prolongierten Reanimationsverlaufs mit ausbleibendem ROSC, anhaltender Asystolie und inzwischen abfallenden etCO_2_-Werten fiel dann die Entscheidung zur Durchführung einer präklinischen perimortalen Crash-Sectio (PPCS) als Ultima Ratio im Sinne eines Rettungsversuches für das ungeborene Kind (+48 min). Parallel wurden ein neonatologisches Team und ein weiteres NEF nachgefordert (aufgrund der langen Anfahrtszeit des neonatologischen Teams).

## Präklinische perimortale Crash-Sectio

Die PPCS erfolgte durch eine mediane Laparotomie (+49 min), wobei die Thoraxkompressionen beim Ansetzen des Skalpells zweimalig für jeweils etwa 5 s unterbrochen werden mussten. Bei Eröffnung des Peritoneums entwichen größere Mengen Luft. Nach vertikaler Stichinzision des Uterus folgte eine bimanuelle stumpfe Vergrößerung der geschaffenen Hysterotomie, durch die das Neugeborene geborgen wurde. Die Nabelschnur wurde geklemmt, mittels Schere durchtrennt und das Neugeborene, wie im Team-Briefing besprochen, unter laufender Reanimation zum vorbereiteten Behandlungsraum in ca. 5 m Entfernung verbracht. Die Gesamtdauer vom Hautschnitt bis Durchtrennung der Nabelschnur betrug 60–90 s ohne Auftreten chirurgischer Komplikationen.

Die weiteren Reanimationsmaßnahmen bei der Mutter wurden schließlich nach 50 min Reanimationsdauer mit kumulativer Gabe von 8 mg Adrenalin und 2500 ml Vollelektrolytlösung bei persistierender Asystolie, auch nach abdomineller Entlastung, beendet, und es erfolgten die Ressourcenbündelung und Konzentration auf die Reanimation des Neugeborenen (+51 min).

## Neugeborenenreanimation

Unmittelbar nach der Entbindung zeigte sich ein schlaffes und zyanotisches weibliches Neugeborenes ohne Lebenszeichen mit initialem Apgar-Score von 0. Unter Reanimation im Rhythmus 3:1 wurden nun mehrere i.o.-Zugänge etabliert (+51 min): zunächst in den linken Humeruskopf (im weiteren Verlauf disloziert), dann in das distale Femur linksseitig von ventral (suffizient), schließlich femoral rechtsseitig (disloziert) und im Humeruskopf rechtsseitig (suffizient). Die i.o.-Zugänge mussten kontinuierlich manuell fixiert werden, da die vorgesehene Klebefixierung bedingt durch Fruchtwasser, Defibrillations- und EKG-Elektroden und die Größe der Extremitäten nicht ausreichte. Der weitere Reanimationsablauf konnte ohne Unterbrechungen fortgesetzt werden. Durch vor Ort befindliche Feuerwehrkräfte wurden Halogenstrahler als Wärmequelle aufgebaut.

Nach 25 min andauernder Neugeborenenreanimation traf das zusätzlich alarmierte NEF ein; der Notarzt verfügte hier (zufällig) über eine weitgehende kinderanästhesiologische Expertise (+77 min). Weiterhin trat einmalig Kammerflimmern mit umgehender externer Defibrillation (20 J, biphasisch) auf, anschließend zeigte sich elektrokardiographisch kurzzeitig eine Eigenfrequenz von 70/min mit sinkender Tendenz. Inwieweit es sich hierbei um eine pulslose elektrische Aktivität (PEA) oder eine Herzaktivität mit Auswurf handelte, ließ sich aufgrund der vorbeschrieben erschwerten Verhältnisse nicht verifizieren, sodass die Reanimation bei Bradykardie und mutmaßlicher PEA umgehend fortgeführt wurde. Nach kurzer Zeit persistierte eine Asystolie.

Nach 40 min andauernden Reanimationsbemühungen um das Neugeborene mit 6‑maliger Gabe von jeweils 30 μg Adrenalin, einmaliger Gabe von 60 μg Atropin und kumulativ 200 ml Volumenbolus wurden einvernehmlich mit allen Beteiligten sämtliche Reanimationsmaßnahmen eingestellt (+91 min). Das auf dem Weg befindliche neonatologische Team wurde abbestellt. Es erfolgte unmittelbar im Anschluss ein „hot debriefing“ unter Einbindung aller Beteiligten und des PSNV-Teams (psychosoziale Notfallversorgung), das sich kontinuierlich (ab +45 min) um die Betreuung der Angehörigen und Zeugen gekümmert hatte. In diesem Rahmen wurde eine Anbindung der Einsatzkräfte an das Einsatznachsorgeteam (ENT) der Berliner Feuerwehr sichergestellt. Insgesamt waren ca. 30 Kräfte der Feuerwehr und des Rettungsdienstes unmittelbar in den Einsatz involviert. Anschließend wurden umfangreiche Personalwechsel und Außerdienstnahmen von am Einsatz beteiligtem Personal durchgeführt.

## Einsatznachbesprechung

**Figure 2:**
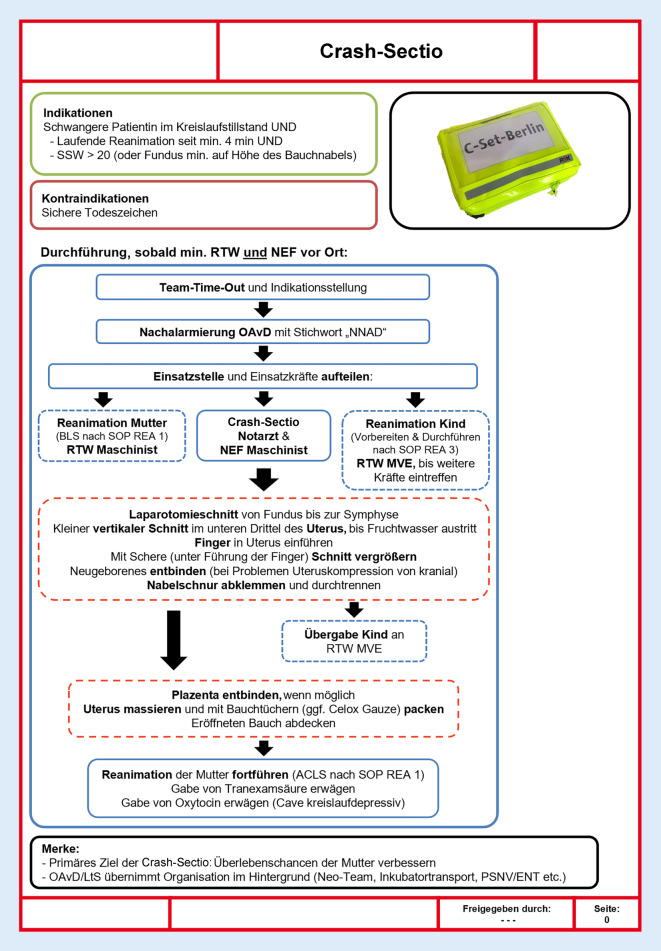

Im zeitlichen Abstand von ca. 2 Wochen fand durch die ärztliche Leitung Rettungsdienst eine strukturierte Einsatznachbesprechung mit nahezu allen beteiligten Kräften vom Notrufsachbearbeiter („call taker“) bis zu den Führungskräften statt, in der die Ereignisse und Erlebnisse aufgearbeitet wurden. Diskutierte kritische Ereignisse sowie die daraus gewonnenen Erkenntnisse zeigt Tab. 1. Als ein weiteres Ergebnis dieser Nachbesprechung wurde aus dem Team heraus an die ärztliche Leitung Rettungsdienst der Wunsch geäußert, eine standardisierte Vorgehensweise mit Beschreibung des Einsatzablaufes für vergleichbare Szenarien in Bezug auf die PPCS zu erstellen. Hieraus wurde in den Folgemonaten der Entwurf einer medizinischen Handlungsanweisung (SOP) entwickelt (Abb. 2).

**Table 1:** 

Bereich	Ereignis/Herausforderung	Ablauf	Bewertung und gewonnene Erkenntnis
Disposition	Beschickung des Einsatzes mit angemessenen Ressourcen und entsprechenden Qualifikationen	Disposition mit initial unzureichendem Einsatzmittelaufgebot nach Einsatzstichwort. Während der Anfahrt zur Einsatzstelle erfolgte die RTH-Nachforderung durch einen RTW. Im Verlauf wurden weitere Ressourcen (neonatologisches Team, ENT) nachgefordert	Die Aktivierung zusätzlicher Ressourcen sollte bereits im Rahmen der Initialdisposition erfolgen. Dies gilt insbesondere im Kontext der bei derartigen Einsätzen benötigten Expertise in maximalinvasiven Maßnahmen. Entsprechende Qualifikationen sind im Land Berlin inzwischen u. a. auf allen luftgebundenen Rettungsmitteln und beim Oberarzt vom Dienst (OAvD) vorhanden. Weiterhin sollten die verfügbaren Ressourcen (Neo-Team) standardisiert und eine zu erwartende Eintreffzeit bekannt sein. Außerdem sollte aufgrund von Vorlaufzeiten frühzeitig ein PSNV/ENT-Team für Angehörige, Umstehende und Rettungskräfte disponiert werden
Kommunikation/Team Resource Management	Team-Time-Out und Entscheidung zu einer maximalinvasiven Intervention	Neben Übergabe und Untersuchung der Patientin mit erweiterter Diagnostik (Sonographie) wurden im Rahmen der Basismaßnahmen noch Zugänge etabliert und Medikamente verabreicht. Zeit von Eintreffen des RTH-Teams bis zum Team-Time-Out mit Entscheidung zur PPCS von 7 min	Bei Eintreffen zusätzlicher medizinischer Kräfte an der Einsatzstelle sollte möglichst sofort eine strukturierte Übergabe erfolgen. Nach Umsetzung sofort erforderlicher Basismaßnahmen sollte unverzüglich ein strukturiertes Team-Time-Out stattfinden, das, falls erforderlich, eine Entscheidung zu einer maximalinvasiven Maßnahme (z. B. nach „FOR-DEC“-Schema) beinhalten kann
Medizinisch-fachlich	Anlage von i.v.- und i.o.-Zugängen bei Patienten mit massivem stumpfen Traumamechanismus	Unter anderem Anlage eines tibialen i.o.-Zugangs	Bei Patienten mit stumpfem Traumaereignis sollte grundsätzlich ein Zugangsweg (i.o. und i.v.) oberhalb des Zwerchfells (einschließlich der oberen Extremitäten) angestrebt werden
	Frühzeitige Entlastung von potenziellen Spannungspneumothoraces als potenzielle reversible Ursache im Rahmen der Traumareanimation	Beidseitige Entlastungspunktion und konsekutive Thoraxdrainagenanlage	Im Rahmen der Traumareanimation ist eine unverzügliche beidseitige Thoraxentlastung unerlässlich. Hierbei sollte, wann immer, möglich die Fingerthorakostomie über die Entlastungspunktion bevorzugt werden, da eine erfolgreiche Durchführung bei der Fingerthorakostomie sicherer verifiziert werden kann. Während der Traumareanimation ist eine vollständige Thoraxdrainagenanlage grundsätzlich nicht notwendig und sollte aus Zeitgründen unterbleiben
	Hämorrhagie als häufige reversible Ursache des traumatischen Kreislaufstillstandes in der Präklinik	Massive Volumengabe im Rahmen der Traumareanimation, die mit (vorgewärmter) kristalloider Infusionslösung durchgeführt wurde	Bei langen Anfahrtszeiten zum nächstgelegenen geeigneten Krankenhaus und massiver Hämorrhagie, die nur unzureichend mit Kristalloiden adressiert werden kann, zeigt sich die Notwendigkeit präklinischer Blutprodukte auch im urbanen Raum
	Indikation zur PPCS	Reanimationsdauer von 41 min bis PPCS erfolgte	Bei erfüllten Indikationskriterien sollte eine PPCS so früh wie möglich erfolgen. Die schnelle Zuführung von erforderlichen Ressourcen mit spezieller Expertise sorgt für eine schnelle Durchführung der Intervention. Als „Ultima-Ratio“-Maßnahme und bei in der Literatur dokumentiertem Überleben von Neugeborenen auch nach beträchtlichen Reanimationsdauern stellt eine protrahierte Reanimation, für sich genommen, keine Kontraindikation zur PPCS dar
	Anlage eines i.o.-Zugangs beim Neugeborenen	Fehllage und unzureichende Sicherungsmöglichkeiten der i.o.-Zugänge, die deshalb kontinuierlich durch umstehendes Personal manuell fixiert werden mussten	Nach Anlage eines i.o.-Zugangs bei Neugeborenen besteht aufgrund physiologischer Besonderheiten und oft fehlender Routine eine erhöhte Dislokationsgefahr. Die vorgesehene Klebefixierung war in diesem Fall unzureichend. Medizinisches Personal sollte dieses Problem antizipieren und entsprechend eine manuelle Fixierung ggf. unter Anwendung von Hilfsmitteln vorbereiten sowie möglichst für Redundanzen sorgen
Nachsorge	Belastende Einsätze im Rettungsdienst	Nach Einsatzende wurde die gesamte RTH-Besatzung ausgewechselt und weitere bodengebundene Einsatzmittel wurden außer Dienst gestellt	Bei entsprechend belastenden Einsätzen sollte von der Notwendigkeit ausgegangen werden, alle am Einsatz beteiligten Kräfte außer Dienst zu nehmen und an Einsatznachsorgeteams anzubinden. Inzwischen ist durch das neu erlassene PSNV-Gesetz im Land Berlin sichergestellt, dass auch Angehörige und Zeugen zeitlich über das initiale Notfallereignis hinaus psychosoziale Notfallversorgung über bestehende PSNV-Strukturen erhalten können

*RTH* Rettungshubschrauber, *RTW* Rettungswagen, *ENT* Einsatznachsorgeteam, *PSNV* psychosoziale Notfallversorgung, *i.o.* intraossär, *i.v.* intravenös

## Diskussion

Die perimortale Crash-Sectio (PMCS) wird in den aktuellen ERC-Leitlinien 2021 beim Kreislaufstillstand in der Schwangerschaft, basierend auf wenigen verfügbaren Daten und einem Expertenkonsens, empfohlen. Sie soll dann erwogen werden, wenn die Schwangerschaft mehr als 20 Wochen besteht (oder der Uterus über dem Niveau des Nabels tastbar ist) und die sofortige Wiederbelebung in den ersten 4 min erfolglos ist. Die PMCS soll möglichst am Ort des Kreislaufstillstands stattfinden. Weiterhin sollte möglichst bereits vor Ablauf der 4 min mit den Vorbereitungen begonnen werden [11]. Manche Autoren fordern jedoch eine Aufhebung dieser „4-Minuten“-Regel und sprechen sich für eine unverzügliche Entbindung nach Eintritt eines Kreislaufstillstands aus [2, 15]. Es wird u. a. zugrunde gelegt, dass im Ergebnis der Entbindung die aortokavale Kompression vermindert wird und somit die Wahrscheinlichkeit einer erfolgreichen Wiederbelebung von Mutter und Kind verbessert wird. Gerade bei Nothysterektomien, die aufgrund eines mütterlichen Herz-Kreislauf-Stillstandes im Kreißsaal stattfanden, wurde wiederholt über ein gutes Überleben von Mutter und Kind berichtet [7]. Dennoch wird als primäres Ziel der Maßnahme regelhaft die Steigerung der Überlebenschance der Mutter betrachtet [15]. Nur unter besonderen Umständen (z. B. Verletzungen der Mutter, die nicht mit dem Leben vereinbar sind) wird der Fokus auf eine Rettung des ungeborenen Kindes gelegt. Erfolgt die Entbindung des Kindes innerhalb von 5 min nach mütterlichem Kreislaufstillstand, wird die Überlebensrate des Neugeborenen in der Literatur teilweise mit 96 % angegeben [1]. Hierbei spielt neben der Zeit zwischen dem Kreislaufstillstand und dem Beginn der Maßnahme eine Reihe weiterer Faktoren eine wesentliche Rolle. Diese sind neben der Ursache des Kreislaufstillstands (z. B. traumatisch vs. internistisch) die Örtlichkeiten sowie die Expertise im Team. Des Weiteren müssen auch die Fähigkeiten und Kompetenzen im Team im Verhältnis zur Entfernung zum nächstgelegenen geeigneten Krankenhaus miteinander abgewogen werden, um bestmögliche Bedingungen für die Durchführung der Maßnahme zu erreichen [14]. Im geschilderten Fall stellte der Transport in die weit entfernte Klinik keine Option dar. Generell darf nach Ansicht der Autoren der Zeitbedarf für einen Transport in eine Klinik nicht unterschätzt werden und ist auch im städtischen Ballungsraum selten kurz genug.

Die tatsächliche Häufigkeit der perimortalen Sectio, insbesondere im prähospitalen Bereich, (PPCS) ist schwer einzuschätzen. Neurologisch intaktes Überleben des Kindes ist in der Literatur auch nach langen Reanimationszeiten und stumpfem Traumaereignis als Ursache des maternalen Kreislaufstillstands beschrieben [4]. Yildirim et al. berichten in einem Fallbericht über eine innerklinische perimortale Crash-Sectio in der Notaufnahme nach 45 min frustraner Reanimation bei mütterlichem penetrierendem Trauma mit neurologisch intaktem Überleben des Kindes im Alter von 6 Monaten [20]. Im Jahr 2014 haben Gatti et al. in einer Kasuistik den Fall einer PPCS bei traumatischem Kreislaufstillstand der Mutter beschrieben. Die Mutter konnte in diesem Fall nicht wiederbelebt werden, aber das Kind überlebte mit nur leichtem neurologischen Defizit [9]. Zwei Fallberichte von Tommila et al. stellen ein Überleben ohne neurologischen Schaden zweier Neugeborener nach PPCS in der 30 + 4 und 26 + 5 SSW dar [17]. Woods beschreibt den Fall einer in der 30. SSW schwangeren Patientin mit plötzlichem Kreislaufstillstand in der Häuslichkeit, bei der 31 min nach beobachtetem Kollaps eine PPCS durchgeführt wurde [19]. Zwei Jahre später hat das damals durch die PPCS gerettete Kind keine feststellbaren neurologischen Schäden. Weitere erfolgreiche Fallberichte gibt es sowohl 45 als auch 15 min nach dem Kollaps der Mutter, wobei es sich bei beiden Fällen um einen traumatischen Kreislaufstillstand handelte (37. und 31. SSW) [10].

In Berlin werden regelmäßig im Rahmen eines „Clinical Forensic Correlation Meeting“ prähospital Verstorbene, die rechtsmedizinisch untersucht wurden, zwischen der ärztlichen Leitung Rettungsdienst und der Berliner Rechtsmedizin nachbesprochen. Die Obduktionsergebnisse im vorliegenden Fall ergaben ein multiples schweres Verletzungsmuster, bei denen mehrere Verletzungen, für sich genommen, und insbesondere in Summe, ex post betrachtet, bei beiden Patientinnen nicht mit dem Leben vereinbar waren. Dies hätte durch die Rettungskräfte vor Ort mit den ihnen zur Verfügung stehenden Mitteln nicht erkannt werden können. Die medizinischen Interventionen waren korrekt ausgeführt worden und die Lage der iatrogen eingebrachten Fremdkörper regelrecht.

Auch wenn in der Literatur vereinzelt dargestellt wird, dass die perimortale Sectio caesarea für die prähospitale Situation keine Rolle spielt [18] und der dargestellte Fall aufgrund verschiedener Faktoren nicht zum Überleben der Mutter oder des Neugeborenen führte, sind die Autoren dennoch davon überzeugt, dass eine derartige Intervention im Rettungsdienst durchaus im Einzelfall zum Versuch der Lebensrettung zum Einsatz kommen kann. Für den Zeitraum, bis eine PPCS (z. B. bei fehlendem Personal) durchgeführt werden kann, gilt: „Save the mother to save the child.“

Da es sich bei der PMCS bzw. PPCS jedoch um sehr seltene Ereignisse handelt, sollten entsprechende Protokolle und Algorithmen vorhanden sein, die im Notfall einfach zugänglich und verständlich sind [6]. Handlungsanweisungen und Checklisten sind gerade in seltenen Notfallsituationen von großer Bedeutung [5]. Auch wenn in den gängigen Kursen für invasive Notfalltechniken eine Vielzahl von Maßnahmen betrachtet wird, ist die PMCS bzw. PPCS bislang meist kein Gegenstand. Eine Integration in diese Kursformate oder in gängige Simulationskurse für geburtshilfliche Notfälle wäre aus Sicht der Autoren durchaus denkbar.

Die durch diesen Fall erworbenen Erkenntnisse, insbesondere zur Vorbereitung solcher Einsatzszenarien, bilden eine wesentliche Voraussetzung sowohl für eine leitlinienadhärente Versorgung im Einsatz als auch eine adäquate Nachsorge der Teammitglieder. Dies bezieht sich vor allem auf gemeinsame mentale Modelle und Vorsorge durch eine umfassende standardisierte Alarmierung von Einsatzmitteln, einheitliche SOP, Schulungen und Trainings, sowie entsprechende materielle Ausstattung. Weitere Ressourcen zu diesem Thema sind in der Infobox zu finden.

### Infobox Ressourcen

Video des bei Greater Sydney Area HEMS seit vielen Jahren ins Einarbeitungskonzept integrierten Workshops zur PPCS: https://sydneyhems.com/2015/08/18/resuscitative-hysterotomy/
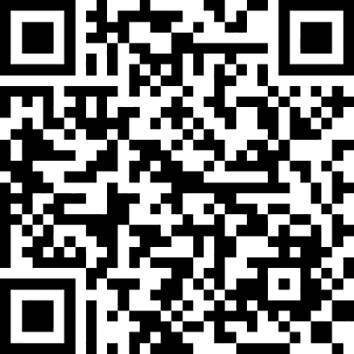


Durchführung einer PMCS während einer Notfallsimulation auf dem SMACC-Kongress 2019: https://youtu.be/HLmxDP20mvk?t=383
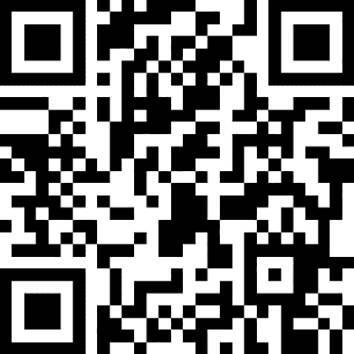


Anleitung zur einfachen „Aufrüstung“ von medizinischen Simulationspuppen, um die PMCS trainieren zu können: https://www.greatnorthairambulance.co.uk/our-work/news/step-by-step-guide-to-creating-a-resuscitative-hysterotomy-manikin/
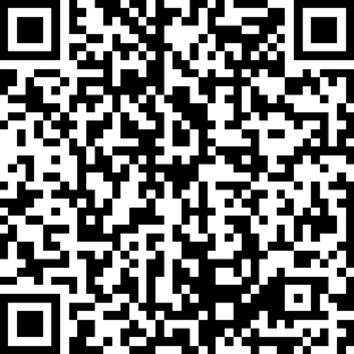


## Fazit für die Praxis

- Die perimortale Crash-Sectio ist laut ERC-Leitlinie indiziert ab der 20. SSW oder bei tastbarem Uterus oberhalb des Bauchnabels nach 4 min laufender kardiopulmonaler Reanimation.
- Primäres Ziel der Maßnahme ist eine Steigerung der mütterlichen Überlebenschancen.
- Überleben des Kindes ist bis 45 min nach mütterlichem Kreislaufstillstand dokumentiert.
- Ein bereits im Voraus systematisch erarbeitetes mentales Modell hilft im Einsatz, kognitive Ressourcen zu schonen.
- Bei psychisch belastenden Einsätzen sind eine frühzeitige Anbindung an Einsatznachsorge-Teams, umfassendes Debriefing und Einsatznachbereitung unerlässlich.
